# Genetic determinants of clinical heterogeneity of the coronary artery disease in the population of Hyderabad, India

**DOI:** 10.1186/s40246-017-0099-1

**Published:** 2017-03-04

**Authors:** Rayabarapu Pranavchand, Arramraju Sreenivas Kumar, Battini Mohan Reddy

**Affiliations:** 1 Molecular Anthropology Group, Biological Anthropology Unit, Indian Statistical Institute, Hyderabad, India; 2 Department of Cardiology, Care Hospitals, Banjara Hills, Hyderabad, India

**Keywords:** Coronary artery disease, Clinical heterogeneity, Genetic association, Pleiotropy, Epistasis

## Abstract

**Background:**

Genetic predisposition to the clinical categories of coronary artery disease (anatomical viz., insignificant, single, double, and triple vessel diseases and phenotypic severity categories viz., angina, acute coronary syndrome, and myocardial infarction) is poorly understood. Particularly, the apolipoprotein genes clustered at 11q23.3 chromosomal region play a vital role in cholesterol homeostasis, and a large number of SNPs identified in this region need to be explored for their association with the clinical categories of CAD.

**Methods:**

Using fluidigm SNP genotyping platform, a prioritized set of 96 SNPs of 11q23.3 chromosomal region were genotyped on 508 CAD cases and 516 ethnicity matched controls, enrolled from Hyderabad, India, and its vicinity.

**Results:**

The association analysis suggests 19 and 15 SNPs to be significantly associated (*p* ≤ 0.05) with at least one of the anatomical and/or phenotypic severity categories, respectively. Overall, the six SNPs rs17440396:G>A, rs6589566:A>G, rs2849165:G>A, rs10488699:G>A, rs1263163:G>A, and rs1263171:G>A were significant even after correction for multiple testing. Three of these (rs17440396:G>A, rs6589566:A>G, and rs2849165:G>A) that belong to *BUD13*, *ZPR1*, and *APOA5-APOA4* intergenic regions, respectively, were found to be associated across the anatomical categories of CAD. However, no particular trend in the genotypic odds ratios with the increasing severity was apparent. The association analysis of the variants with phenotypic severity categories suggests that a high degree of phenotypic severity could be a result of more number of risk alleles. While the risk score analysis suggests high discriminative power of the variants towards the individual clinical categories of CAD, the complex network of interactions seen between the intronic variants of *BUD13* and *ZPR1* regulatory genes and intergenic variants of *APOA5-APOA4* suggests pleiotropic effects of regulatory genes in the manifestation of these CAD categories.

**Conclusion:**

The complex network of interactions observed in the present study between the regulatory and protein-coding genes suggests their role in the manifestation of distinct clinical categories of CAD, which needs to be functionally validated.

**Electronic supplementary material:**

The online version of this article (doi:10.1186/s40246-017-0099-1) contains supplementary material, which is available to authorized users.

## Background

Coronary artery disease (CAD) results from a progressive damage of blood vessels that supply blood to the heart muscle. It is caused by a process of hardening of arteries called atherosclerosis, which involves both genetic and environmental factors and interaction between them. A large number of genes that belong to lipoprotein metabolism are found associated with monogenic and polygenic forms of CAD [1]. Particularly, *APOA1*, *APOC3*, *APOA4*, and *APOA5* genes clustered in 11q23.3 human chromosomal region are predominantly expressed in the liver and intestine and crucial in regulating lipoprotein metabolism and cholesterol homeostasis [2]. However, several variants that belong to this apolipoprotein gene cluster region were found to be associated with increased risk towards overall CAD phenotype and/or elevated lipid traits among the Indians [3–6]. Our recent study based on the 96 SNPs from this region suggested distinct patterns of association of these variants with CAD and dyslipidemia. This pleiotropic nature of association was explained by the observed SNP-SNP interactions between regulatory and apolipoprotein coding genes [6].

CAD is identified as a broad phenotype that manifests as less severe stable angina (SA) or unstable angina (UA) and more severe forms like acute coronary syndrome (ACS) and myocardial infarction (MI). Further, the advanced coronary angiography modalities classify CAD cases into distinct anatomical categories depending upon the number of vessels and length and diameter of the atherosclerotic lesions in the coronary blood vessels. However, defining CAD cases is difficult due to the process of multi-decadal atherosclerotic phenomenon that can manifest any of the CAD conditions. With the characteristic clinical heterogeneity and the difficulty in defining CAD cases, it has become a challenging trait to study [7]. There was substantial phenotypic heterogeneity of the samples hitherto considered for genetic association studies and that might be one of the reasons for the lack of consistency in the findings of those studies [8]. The couple of studies on the genetic predisposition to anatomical categories were restricted to 9p21.3 locus among the Caucasians [9, 10], except for an increasing genotypic odds ratio observed with increasing number of diseased blood vessels in case of a single polymorphism of the *APOA1* gene (−75G>A (*MspI*)) in two independent studies, among the north Indian [11] and Australian populations [12]. Despite the vital role in the process of atherosclerosis envisaged for the apolipoprotein genes clustered at 11q23.3 chromosomal region, no further attempts were made to understand their associations with anatomical or phenotypic severity categories of CAD. Given the characteristic dyslipidemic feature of Indian populations in general and particularly of the southern Indians, it is pertinent to explore the pattern of association of the variants of this genomic region with each of the anatomical and phenotypic severity categories of CAD, which might throw light on the possible genetic mechanisms responsible for progression of the disease. We present here the results of our association analysis of a prioritized set of 96 SNPs at 11q23.3 region with sub phenotypes of CAD classified according to anatomical and phenotypic severity.

## Methods

### The study design and population

The population of Hyderabad is a conglomeration of people from different parts of the undivided state of Andhra Pradesh, and the mother tongue of most of its populations is Telugu, one of the four Dravidian languages. It would be also pertinent to note that despite the subdivision of Telugu population into a number of traditionally endogamous castes and subcastes, Reddy et al. [13] observed genetic differentiation among the populations of Andhra Pradesh to be very low and insignificant; the Markov chain Monte Carlo analysis of population structure, which implements model-based clustering method for grouping individuals into populations, did not reveal any unique population clusters, suggesting high degree of genetic homogeneity.

A total of 1024 individuals, including 508 CAD cases and 516 controls, representing the population of Hyderabad, participated in our case-control study. Patients with characteristic symptoms of stable/unstable angina pectoris along with varying degrees (generally >40%) of stenosis in at least one of the major coronary arteries as determined through angiogram were included in the study. Cases with monogenic diseases, valvular heart disease, cardiomyopathy, renal disease, acute and chronic viral or bacterial infections, asthma, tumors or connective tissue diseases, and other vascular diseases were excluded from the study. All the cases were recruited at the CARE Hospitals, Hyderabad, and evaluated by interventional cardiologists for the above mentioned criteria. Control samples were recruited by conducting free health camps in and around Hyderabad, mostly representing subjects aged above 45 years and with similar ethnic backgrounds as that of the cases. The individuals with characteristic features of any of the above mentioned disease conditions were not included as part of the controls. However, certain proportions of both the case and control subjects were found with T2DM, dyslipidemia, and hypertension.

### Data and sample collection

Data pertaining to present age, sex, and age at diagnosis for cases and other background information such as history and current status of smoking, alcoholism, and food habits were obtained through a detailed questionnaire. Information regarding the current status of the subjects on diabetes, dyslipidemia, and hypertension were drawn from hospital records for the cases and through personal interviews for the controls. About 5–6 ml of fasting blood sample was collected peripherally by certified medical lab technicians. Clinical investigations were done for lipid profile and blood sugar for all the samples at Tapadia Diagnostic Centre, Hyderabad, using Auto Analyzer. Blood pressure along with height, weight, waist circumference, and hip circumference were also measured in the field for all the controls, and for the cases, these data were obtained from hospital records.

### DNA isolation, SNP selection, and genotyping

DNAs were isolated from all the samples using phenol chloroform method [14] and quantified with the help of Thermo Scientific Varioskan™ Flash Multimode Reader using Quant-iT™ PicoGreen® dsDNA Assay Kit. In order to comprehensively genotype the variants at 11q23.3 chromosomal region, we gathered information on SNPs pertaining to this region from earlier candidate gene and sequencing studies and from databases particularly EBI-NHGRI GWAS database, HAPMAP and dbSNP. Given the key role of *BUD13* in splicing mechanism and *ZPR1* as essential protein for normal cell proliferation and signal transduction, in addition to the SNPs of *APOAI-CIII-AIV-AV* genes clustered at 11q23.3 chromosomal region, we also included SNPs related to these regulatory protein-coding genes. A total of 130 SNPs, studied through candidate gene and GWAS approaches, were subjected to Fluidigm D3 Assay design software [15], and a panel of 96 SNPs with high efficiency for genotyping was chosen. Genotyping was performed using fluidigm nanofluidic SNP genotyping system. Eleven 96.96 IFC chips were utilized for genotyping wherein the selected 96 SNPs were analyzed against 96 samples in each chip. These chips were thermal cycled, and the endpoint fluorescent values were measured on Biomark™ system. Final sample wise genotype calls were obtained using Fluidigm SNP Genotyping Analysis software. A subset of 240 samples was genotyped prior to genotyping of the total 1024 samples. The observed call concordance was 100%.

Prior to genetic association analysis, data quality control was achieved by limiting the sample wise call rate to ≥90%. This resulted in a genotype call rate of 99% in 386 cases and 462 controls, which were considered for further analysis. Further, after excluding SNPs that showed either minor allele frequency <1% and/or deviated from hardy Weinberg equilibrium (*p* < 0.001), only 75 of the 96 SNPs were qualified for final analysis.

### Clinical categories of the CAD cases

The CAD cases were categorized into the following four anatomical sub types : (i) cases with 40–70% stenosis and symptomatic for CAD with characteristic atherosclerotic lesions as “insignificant disease,” (ii) with >70% stenosis in any one of the major coronary blood vessel as “single vessel disease (SVD),” (iii) with >70% stenosis in two major coronary blood vessels as “double vessel disease (DVD),” and (iv) with >70% stenosis in three major coronary blood vessels are categorized as “triple vessel disease (TVD).” After the genotype pruning, 93 cases with insignificant stenosis, 121 with SVD, 75 with DVD, and 70 with TVD remained with approximately 99% genotype call rate. We also categorized the cases based on the phenotypic severity into three broad classes, (i) those with characteristic symptoms of stable or unstable angina (SA/US), (ii) with symptoms of acute coronary syndrome (ACS), and (iii) with reported myocardial infarction (MI). After the genotype pruning, 73 cases with SA/UA, 165 with ACS, and 76 with MI remained with approximately 99% genotype call rate. The cases that could not be assigned to any of the anatomical/phenotypic severity categories were excluded from the association analysis.

### Statistical methods

The descriptive statistical analysis of the background data on quantitative variables was done using MINITAB (version 17). Genotyping quality check and association analysis of alleles were done using PLINK [16]. Genotype-phenotype association analysis using logistic regression assuming different genetic models-dominant, co-dominant, recessive, over dominant, and log-additive were performed using “SNPassoc” package of R PROGRAM [17].

## Results and discussion

The means and standard deviations of quantitative parameters along with *t* values for the difference in means of controls and each of the anatomical and phenotypic severity categories are provided in Additional files 1 and 2, respectively. The average age of individuals of the TVD category is significantly higher than that of controls as well as other less severe anatomical categories. Analysis of variance suggests significant heterogeneity in the mean age of subjects among the anatomical categories, which is because of the difference between the average age of TVD and all other anatomical categories. When compared to controls, a significant increase in the means of fasting blood sugar (FBS) and decrease in mean high density lipoprotein cholesterol (HDLC) and the low density lipoprotein cholesterol (LDLC) levels were observed in each of the anatomical and phenotypic severity categories. The elevated levels of triglycerides (TG) and very low density lipoproteins (VLDL) were observed for the SVD group in comparison to the other anatomical categories and for the ACS in comparison to other phenotypic severity categories.

### Association of variants at 11q23.3 chromosomal region with anatomical categories of CAD

The test of association of variants at the 11q23.3 chromosomal region yielded 19 SNPs to be significantly associated (*p* ≤ 0.05) with at least one of the four anatomical categories of CAD which clearly implicates the potential role of these variants in the manifestation of these categories. The minor allele frequencies of the associated SNPs with respective odds ratios are presented in Table 1. Seven, ten, nine, and 11 of these 19 SNPs are observed to be associated with insignificant, SVD, DVD, and TVD categories, respectively. However, while the risk-reducing effect of the intronic *BUD13* variant rs17440396:G>A and two *APOA5-APOA4* intergenic variants rs633389:C>T and rs2849165:G>A is consistent across the anatomical categories, rs6589566:A>G of *ZPR1* gene is consistently associated with increased risk. Of the remaining three SNPs associated with insignificant category, rs10488699:G>A and rs625524:G>A confer risk and rs3741298:A>G is protective. Of the remaining six of the nine SNPs associated with SVD, rs664059:C>T, rs2075294:G>T, rs633867:C>T, rs5081:A>T, and rs632153:G>T are associated with increased risk while only rs5072:C>T is associated with decreased risk. Among the five remaining SNPs associated with DVD, while rs1263163:G>A and rs7396835:C>T are associated with decreased risk rs1263171:G>A, rs2727793:G>A, and rs2542063:G>A are with increased risk towards the condition. Similarly, of the remaining seven SNPs associated with TVD, while rs10488699:G>A, rs664059:C>T, rs2075294:G>T, and rs1263171:G>A confer risk rs11600380:T>C, rs1263163:G>A, and rs625524:G>A are protective in nature. We applied a powerful yet less stringent Benjamin-Hochberg (BH) correction for multiple testing and found rs17440396:G>A to be highly significantly associated across the anatomical categories, while the two SNPs, rs6589566:A>G and rs2849165:G>A, remained significantly associated with insignificant, SVD, and DVD categories. The results of genotypic association analysis of the above SNPs with anatomical categories under the log-additive model (Additional file 3) suggest a similar pattern of association of the SNPs as in the case of allelic association, except for rs3741298:A>G in case of insignificant category and rs7396835:C>T in case of DVD and both rs664059:C>T and rs2075294:G>T in case of TVD. Among the variants that belong to *BUD13*, *ZPR1*, and *APOA5-APOA4* intergenic regions (rs17440396:G>A, rs6589566:A>G, and rs2849165:G>A), respectively, and found to be associated across the anatomical categories of CAD, no particular trend with increasing severity of the disease was evident in the genotypic odds ratios. The observed pattern of association may thus suggest the implicit pathophysiology of the disease where the abnormal lipid metabolism only triggers the process of atherosclerosis and may not play a major role in further progression of the disease [18]. Further, an increasing genotypic odds ratio of the -75G>A (MspI) polymorphism in the promoter region of *APOA1* gene observed with increasing anatomical severity among the north Indians [11] and in an Australian population [12] could not be observed in our population. Concurrently, the significant association of two variants, rs1225006 (Chromosome 3; *CPNE4* gene) and rs6745588 (Chromosome 2; *STK39* gene), with triple vessel disease discovered in a recent GWAS on Koreans were not associated in a replication cohort [19]. Except for this single GWAS study and a few validating studies on 9p21.3 variants and Msp1 *APOA1* conventional polymorphism, genetic determinants of the clinical heterogeneity of CAD are hitherto poorly understood.

**Table 1:** Association of variants at 11q23.3 chromosomal region with anatomical categories of CAD

SNP (major/minor allele)	Nearby/associated gene	MAF in Controls *N* = 462	Insignificant (*n* = 93)			Single vessel disease (*n* = 121)			Double vessel disease (*n* = 75)			Triple vessel disease (*n* = 70)		
			MAF	*p* value	OR (95% CI)	MAF	*p* value	OR (95% CI)	MAF	*p* value	OR (95% CI)	MAF	*p* value	OR (95% CI)
*rs17440396:G>A*	BUD13	0.21	0.02	3.5 × 10^−10^	0.06 (0.02–0.20)	0.03	1.3 × 10^−10^	0.13 (0.06–0.27)	0.03	2.3 × 10^−07^	0.13 (0.05–0.32)	0.04	2.1 × 10^−06^	0.15 (0.06–0.37)
rs10488699:G>A		0.20	0.26	0.040	1.47 (1.02–2.13)							0.27	0.042	1.55 (1.01–2.35)
rs664059:C>T		0.30				0.38	0.017	1.43 (1.06–1.92)				0.39	0.042	1.47 (1.01–2.13)
*rs6589566:A>G*	ZPR1	0.25	0.43	1.5 × 10^−06^	2.25 (1.61–3.14)	0.37	0.0005	1.71 (1.26–2.33)	0.42	1.9 × 10^−05^	2.18 (1.52–3.13)	0.39	0.002	1.86 (1.25–2.76)
rs3741298:A>G		0.35	0.27	0.038	0.68 (0.48–0.98)									
rs2075294:G>T		0.05				0.10	0.012	1.93 (1.15–3.24)				0.09	0.048	1.89 (1.00–3.59)
rs633389:C>T	APOA5-APOA4	0.16	0.08	0.004	0.44 (0.25–0.78)	0.10	0.033	0.62 (0.39–0.97)	0.07	0.003	0.38 (0.19–0.73)	0.07	0.005	0.38 (0.19–0.76)
rs633867:C>T		0.06				0.10	0.020	1.81 (1.09–2.99)						
rs11600380:T>C		0.31										0.22	0.030	0.62 (0.41–0.96)
rs1263163:G>A		0.21							0.11	0.009	0.50 (0.30–0.85)	0.10	0.004	0.44 (0.25–0.78)
rs625524:G>A		0.05	0.10	0.006	2.21 (1.24–3.92)							0.01	0.028	0.15 (0.02–1.07)
rs1263171:G>A		0.43							0.53	0.029	1.47 (1.04–2.08)	0.55	0.008	1.62 (1.13–2.32)
rs2727793:G>A		0.37							0.45	0.044	1.43 (1.01–2.02)			
rs7396835:C>T		0.44							0.35	0.042	0.69 (0.48–0.99)			
rs2542063:G>A		0.37							0.45	0.047	1.43 (1.00–2.02)			
*rs2849165:G>A*		0.36	0.20	3.1 × 10^−05^	0.45 (0.31–0.66)	0.22	2.9 × 10^−05^	0.49 (0.35–0.69)	0.21	0.0002	0.47 (0.31–0.71)	0.23	0.004	0.54 (0.35–0.83)
rs5081:A>T	APOA1	0.03				0.07	0.008	2.25 (1.22–4.16)						
rs5072:C>T		0.37				0.30	0.037	0.72 (0.53–0.98)						
rs632153:G>T		0.03				0.06	0.020	2.11 (1.11–4.02)						

Italic font—significant after Benjamin Hochberg Correction, blank cell—not significant

*MAF* minor allele frequency, *OR* odds ratio obtained from logistic regression analysis

### Association of variants at 11q23.3 chromosomal region with phenotypic severity categories

The results of allelic association with each of the phenotypic severity categories are presented in Table 2. Overall, 15 SNPs were significantly associated (*p* ≤ 0.05) with at least one of the three phenotypic severity categories. However, only four of the 15 SNPs in case of angina, eight in case of ACS and 11 in case of MI phenotypes were individually associated. Among these 15, only two SNPs, rs17440396:G>A and rs633389:C>T, were commonly associated with decreased risk towards all the three phenotypic severity categories. The remaining two SNPs rs6589566:A>G and rs1263167:A>G associated with angina are risk conferring in nature. Of the six remaining SNPs associated with ACS, while rs2187126:A>G and rs2849165:G>A were associated with decreased risk, rs664059:C>T, rs6589566:A>G, rs2075294:G>T, and rs633867:C>T were associated with increased risk towards the condition. In case of MI, while the seven of the nine remaining SNPs (rs10488699:G>A, rs664059:C>T, rs2075294:G>T, rs1263171:G>A, rs2849176:G>C, rs5081:A>T ,and rs632153:G>T) were found to be risk conferring, rs1263163:G>A and rs2849165:G>A are risk reducing in nature. The observed trend of increasing number of risk conferring SNPs associated with increasing phenotypic severity (angina to MI) imply that the variants at 11q23.3 chromosomal region might regulate the phenotypic severity. However, when non MI-angina cases were used as controls in the analysis, none of these additional risk variants turned out to be significant except for the risk reducing effect of rs1263163:G>A on MI, reflecting its specificity towards MI. After correction for multiple testing, while only rs17440396:G>A exhibited significant association with all the three phenotypic categories, rs6589566:A>G and rs2849165:G>A show significant association with angina and ACS and ACS and MI categories, respectively.

**Table 2:** Association of variants at 11q23.3 chromosomal region with stable/unstable angina, ACS, and MI categories

SNP	Control (*n* = 462)	Angina (*n* = 73)			Acute coronary syndrome (*n* = 165)			Myocardial infarction (*n* = 76)		
	MAF	MAF	*p* value	OR (95% CI)	MAF	*p* value	OR (95% CI)	MAF	*p* value	OR (95% CI)
rs17440396:G>A	0.21	0.01	1.36 × 10^−08^	0.05 (0.01–0.21)	0.03	5.15 × 10^−14^	0.12 (0.06–0.23)	0.03	6.89 × 10^−08^	0.10 (0.04–0.28)
rs10488699:G>A	0.20							0.34	0.0001	2.08 (1.41–3.07)
rs2187126:A>G	0.13				0.09	0.025	0.61 (0.39–0.94)			
rs664059:C>T	0.30				0.37	0.029	1.34 (1.03–1.75)	0.41	0.0080	1.61 (1.13–2.30)
rs6589566:A>G	0.25	0.46	5.63 × 10^−07^	2.56 (1.75–3.73)	0.43	3.36 × 10^−09^	2.23 (1.70–2.92)			
rs2075294:G>T	0.05				0.08	0.049	1.63 (1.00–2.66)	0.10	0.026	1.97 (1.07–3.62)
rs633389:C>T	0.16	0.06	0.0027	0.36 (0.18–0.72)	0.09	0.0054	0.56 (0.37–0.85)	0.07	0.0053	0.41 (0.22–0.78)
rs633867:C>T	0.06				0.09	0.015	1.77 (1.11–2.82)			
rs1263163:G>A	0.20							0.03	1.64 × 10^−08^	0.11 (0.04–0.29)
rs1263167:A>G	0.09	0.15	0.037	1.70 (1.03–2.82)						
rs1263171:G>A	0.43							0.52	0.040	1.44 (1.02–2.03)
rs2849165:G>A	0.36				0.17	1.18 × 10^−10^	0.35 (0.26–0.49)	0.23	0.0013	0.51 (0.34–0.77)
rs2849176:G>C	0.49							0.59	0.032	1.46 (1.03–2.08)
rs5081:A>T	0.03							0.07	0.0203	2.28 (1.12–4.65)
rs632153:G>T	0.03							0.06	0.035	2.18 (1.04–4.59)

Blank cell—not significant

*OR* odds ratio obtained from logistic regression analysis

Except for the three of the above 15 SNPs, rs1263167:A>G, rs2075294:G>T, and rs632153:G>T that failed to show significant association with angina, ACS, and MI categories, respectively, the results of genotypic association with the phenotypic severity categories under the log-additive model suggest similar pattern as that of the allelic association (Additional file 4). Nevertheless, those three SNPs which lost significance were the ones with marginal significance (*p* = ~0.05) in case of allelic association, and these departures can be attributed to chance fluctuations because of small sample sizes.

### Pattern of association of genetic variants with anatomical and phenotypic severity categories of the CAD patients using subsets of controls

The control cohort of the present study constitutes two kinds of subjects: (1) those presenting any one or more of the conditions like dyslipidemia, diabetes, and hypertension and (2) those having none of these conditions. In order to determine the confounding effects of these clinical risk factors, particularly dyslipidemia, we repeated the allelic association analysis by using only nondyslipidemic cohort of controls (cohort 1; *n* = 270) and those devoid of any of the three conditions—dyslipidemia, diabetes and hypertension (cohort 2; *n* = 129) and present here only the salient features of the findings of this exploratory analysis.

Overall, a consistent pattern of association of the SNPs rs17440396:G>A, rs6589566:A>G, rs2849165:G>A, rs10488699:G>A, rs1263163:G>A, and rs1263171:G>A is observed with both anatomical and/or phenotypic severity categories, which needs to be explored further for their functional role. The respective *p* values for these SNPs after BH correction for multiple testing in the three sets of controls (total controls, cohort 1, and cohort 2) are presented in Table 3. Certain inconsistencies observed with reference to SNPs that showed significant association at *p* ≤ 0.05 could be due to sampling errors possibly emanated from reduced sample size for the subsets of control. The salient features of the results are the following:

**Table 3:** *P* values for variants that were significantly associated after Benjamin Hochberg correction for multiple testing

SNP	Control choice	Anatomical category (*p* values)				Phenotypic severity categories (*p* values)		
		Insignificant	SVD	DVD	TVD	ANGINA	ACS	MI
rs17440396:G>A	Total Controls	4.92 × 10^−08^	1.49 × 10^−08^	2.02 × 10^−05^	0.0001	1.43 × 10^−06^	1.04 × 10^−11^	6.79 × 10^−06^
	Cohort 1	2.36 × 10^−06^	2.13 × 10^−06^	0.0005	0.0027	2.54 × 10^−05^	4.78 × 10^−09^	0.0001
	Cohort 2	1.77 × 10^−06^	2.47 × 10^−06^	0.0004	0.0021	2.18 × 10^−05^	1.55 × 10^−08^	0.0001
rs6589566:A>G	Total Controls	0.0001	0.012	0.0008		2.61 × 10^−05^	1.26 × 10^−07^	
	Cohort 1	0.017		0.044		0.004	0.0005	
	Cohort 2					0.036	4.70 × 10^−06^	
rs2849165:G>A	Total Controls	0.001	0.001	0.0093			7.65 × 10^−09^	0.026
	Cohort 1	0.011	0.010	0.044			4.30 × 10^−07^	
	Cohort 2	0.015	0.016				0.020	
rs10488699:G>A	Total Controls							0.004
	Cohort 1							0.0007
	Cohort 2							0.018
rs1263163:G>A	Total Controls							7.83 × 10^−06^
	Cohort 1							0.0033
	Cohort 2							0.011
rs633389:C>T	Total Controls			0.014				
rs5081:A>T	Cohort 1		0.047					
rs633867:C>T	Cohort 1		0.047					
rs632153:G>T	Cohort 1		0.047					
rs1263171:G>A	Cohort 1				0.046			

Blank cell—not significant, total control samples (*n* = 462), cohort 1—nondyslipidemic control cohort (*n* = 270), cohort 2—control cohort devoid of dyslipidemia, diabetes, and hypertension (*n* = 129)

1. The rs17440396:G>A is significantly associated across the anatomical and phenotypic categories, irrespective of control cohorts used in the analysis.
2. The rs6589566:A>G is significantly associated with insignificant, SVD, and DVD categories in the analysis using total controls and insignificant and DVD anatomical categories in cohort 1 analysis. Further, this variant is associated with angina and ACS phenotypic severity categories irrespective of control cohorts used in the analysis.
3. The rs2849165:G>A is significantly associated with insignificant, SVD, and ACS categories irrespective of the control cohort used. However, it is associated with DVD in the analysis using total controls or cohort 1 and MI using total controls.
4. The rs10488699:G>A and rs1263163:G>A variants are found associated with MI irrespective of control cohorts used in the analysis.

While rs633389:C>T is significantly associated with DVD when total controls were used, rs5081:A>T, rs633867:C>T, and rs632153:G>T showed significant association with SVD and rs1263171:G>A with TVD in case of control cohort 1.

### Results of SNP-SNP interaction analysis

While the rs17440396:G>A of *BUD13* gene showed risk reducing effects, rs10488699:G>A variant that belongs to the same gene is associated with increased risk particularly for severe forms of anatomical and phenotypic conditions. Similarly, rs3741298:A>G that belong to ZPR1 gene is associated with risk-reducing effect specific to the insignificant category despite the high risk conferring nature towards CAD due to rs6589566:A>G variant. Therefore, we performed pair wise logistic regression analysis to understand the interaction effects of these SNPs on the anatomical and phenotypic categories of CAD. From the pairs of SNPs that showed significant interactions, the following types of interactions can be inferred:

1. Interactions between intronic SNPs of *BUD13* regulatory gene and intergenic SNPs of *APOA5*-*APOA4* genes.
2. Intronic SNPs of *ZPR1* and *BUD13* genes.
3. Intronic SNPs of *ZPR1* gene and Intergenic SNPs of *APOA5*-*APOA4*.
4. Interactions among the variants of *APOA5*-*APOA4* intergenic region.

The pair wise SNP-SNP interactions associated with anatomical categories of CAD are presented in Table 4. The interaction odds ratio of SNPs suggests epistatic effects of rs3741298:A>G over rs6589566:A>G resulting in reducing risk for SVD. While rs2187126:A>G–rs1263171:G>A (OR = 6.07, *p* value = 3.81 × 10^−06^) that is characteristic of type 1 interaction is associated with increased risk towards SVD, rs6589566:A>G–rs1263163:G>A (OR = 5.39, *p* = 1.55 × 10^−05^) of type 3 interaction showed increased risk towards insignificant category. Most of the interactions of variants of *BUD13* with variants of *APOA4*-*APOA5* intergenic region exhibit risk reducing effects to SVD. Further, a type 4 SNP-SNP interaction, rs1263163:G>A–rs2849165:G>A is observed to exhibit risk reducing effects across the anatomical categories while the type 2 interaction, namely, rs6589566:A>G–rs3741298:A>G, showed similar effect only towards SVD.

**Table 4:** Significant SNP-SNP interaction odds ratios from pair wise logistic regression with anatomical categories

Type of interaction	SNP pair	Associated category	Odds ratio	*p* value
BUD13–intergenic variants of APOA5-APOA4 genes	rs10488699:G>A–rs1263163:G>A	Insignificant	0.13	4.43 × 10^−06^
	rs2187126:A>G–rs1263163:G>A	Insignificant	0.04	3.11 × 10^−06^
	rs10488699:G>A–rs1263163:G>A	SVD	0.06	2.26 × 10^−07^
	rs2187126:A>G–rs633389:C>T	SVD	0.06	8.28 × 10^−07^
	rs2187126:A>G–rs1263163:G>A	SVD	0.02	3.00 × 10^−07^
	rs2187126:A>G–rs1263171:G>A	SVD	6.07	3.81 × 10^−06^
Intronic variants of ZPR1–BUD13 genes	rs6589566:A>G–rs3741298:A>G	SVD	0.29	1.36 × 10^−05^
ZPR1–intergenic variants of APOA5-APOA4 genes	rs6589566:A>G–rs1263163:G>A	Insignificant	5.39	1.55 × 10^−05^
Within the intergenic variants of APOA5-APOA4 genes	rs1263163:G>A–rs2849165:G>A	Insignificant	0.03	1.12 × 10^−10^
		SVD	0.06	7.70 × 10^−09^
		DVD	0.03	1.90 × 10^−07^
		TVD	0.06	9.06 × 10^−06^

The interaction analysis with phenotypic severity categories suggests significant SNP-SNP interactions towards angina and ACS categories but not with MI category. Pertaining to angina, only two SNP pairs, namely, rs2187126:A>G–rs1263163:G>A (odds ratio = 0.01; *p* value = 6.4 × 10^−05^) and rs1263163:G>A–rs2849165:G>A (odds ratio = 0.03; *p* = 1.5 × 10^−09^) characteristic of type 1 and type 2 interactions, respectively, showed significant risk-reducing effects towards the condition. The interactions associated with ACS (Table 5) are of type 1, and type 4, and confer significant risk-reducing effects towards the condition. Further, eight SNPs of *BUD13* gene individually showed significant interaction with rs6589566:A>G of ZPR1 gene with six of these interactions being risk conferring towards the ACS (Table 5). Besides this, the interaction of rs6589566:A>G with an APOA5-APOA4 intergenic variant rs1263163:G>A is also risk conferring towards ACS.

**Table 5:** Significant SNP-SNP interaction odds ratios from pair wise logistic regression with acute coronary syndrome^a^

Type of interaction	SNP pair		Odds ratio	*p* value
BUD13–intergenic variants of APOA5-APOA4 genes	rs10488699:G>A	rs633389:C>T	0.17	1.48 × 10^−05^
	rs10488699:G>A	rs1263163:G>A	0.10	9.82 × 10^−09^
	rs2187126:A>G	rs633389:C>T	0.04	1.95 × 10^−07^
	rs2187126:A>G	rs1263163:G>A	0.06	5.17 × 10^−08^
Intronic variants of BUD13–ZPR1–genes	rs11216126:A>C	rs6589566:A>G	2.62	2.11 × 10^−05^
	rs11216129:C>A		2.48	8.22 × 10^−05^
	rs180326:A>G		0.36	5.61 × 10^−05^
	rs2075295:T>C		2.76	2.46 × 10^−06^
	rs17119975:T>C		2.66	1.71 × 10^−05^
	rs1263149:A>G		0.34	1.78 × 10^−06^
	rs623908:A>G		2.39	4.17 × 10^−05^
	rs2041967:A>G		2.69	4.67 × 10^−06^
ZPR1–intergenic variants of APOA5-APOA4 genes	rs6589566:A>G	rs1263163:G>A	4.31	4.59 × 10^−06^
Within the intergenic variants of APOA5-APOA4 genes	rs1263163:G>A	rs1263171:G>A	0.35	2.87 × 10^−05^
	rs1263163:G>A	rs2849165:G>A	0.04	2.85 × 10^−12^

^a^No SNP-SNP interaction was significant in case of angina and MI

The observed trait specific associations might be due to the pleiotropic nature of *BUD13* and *ZPR1* regulatory genes and their interaction with variants at *APOA5-APOA4* intergenic region. Being the component of retention and spliceosome (RES) complex, *BUD13* protein acts as splicing factor for a number of genes and is involved in controlling pre-mRNA retention in the nucleus [20, 21]. *ZPR1* is another prominent pleiotropic regulatory gene whose deficiency causes defects in DNA replication, transcription, and cell cycle regulation [22]. However, the putative pleiotropic effects of these genes in atherosclerosis need to be validated through in vitro functional experiments. Although the present study included (i) missense and nonsense variants and (ii) variants in the noncoding regions such as promoters, 5′ or 3′ untranslated regions (UTR), upstream and downstream elements, and intergenic regions, we observed the intronic SNPs of BUD13 and ZPR1 genes and intergenic SNPs of APOA5-APOA4 genes to be mostly associated with CAD and/or its categories. In fact, most of the SNPs that belong to this region and associated with abnormal lipid traits through GWAS approach are localized to noncoding domains, which has also been the characteristic pattern of association of the genomic regions with other complex genetic diseases. Further, the candidate gene association studies for risk factors of CAD observed several conventional polymorphisms such as 3238 C>G Sac1 (3′UTR SNP of APOC3 gene), −1131 C>T (promoter SNP of APOA5 gene), −75G>A, and +83C>T (promoter SNPs of APOA1 gene) that belong to the noncoding domains of this genomic region [3, 4, 23, 24]. The outcome of ENCODE project also suggests that the noncoding variants may be involved in regulation of gene expression depending on the cell type, developmental stage, and environmental factors [25, 26] and thus have multiple targets. Prioritization of intergenic SNPs through computational tools and subsequent validation through expression GWAS presents a promising strategy for understanding the underlying mechanisms of CAD [27]. For example, Musunuru et al. [28], identified a single noncoding DNA variant rs12740374 at 1p13.3 chromosomal region to influence LDLC and MI risk via liver specific transcriptional regulation of the SORT1 gene by C/EBP transcription factors, providing insights into mechanisms by which the noncoding genetic variants can lead to clinical phenotypes.

### Risk score analysis

**Fig. 1:**
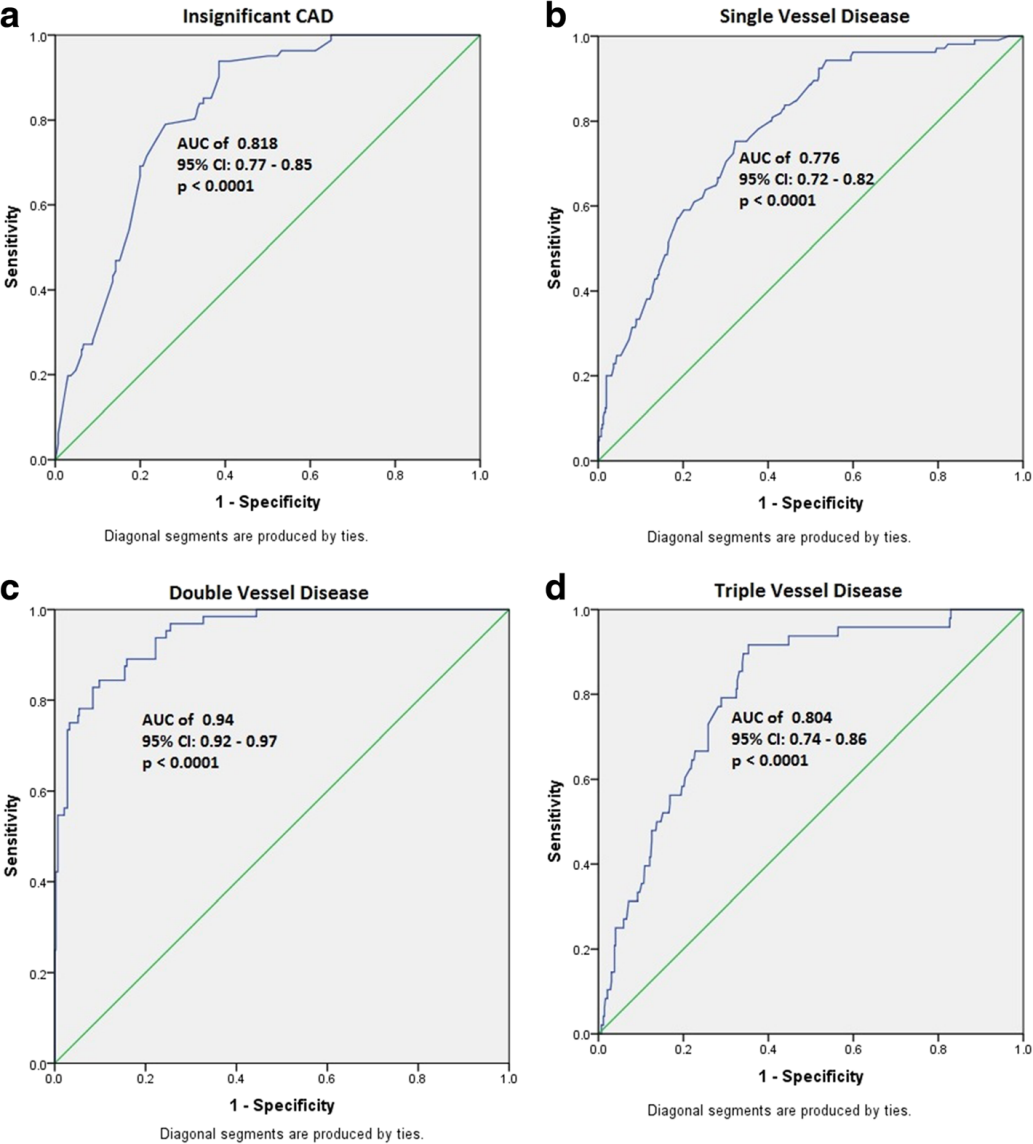
ROC analysis indicating discriminative power of the variants for anatomical categories of CAD. **a** Insignificant CAD. **b** Single vessel disease. **c** Double vessel disease. **d** Triple vessel disease

**Fig. 2:**
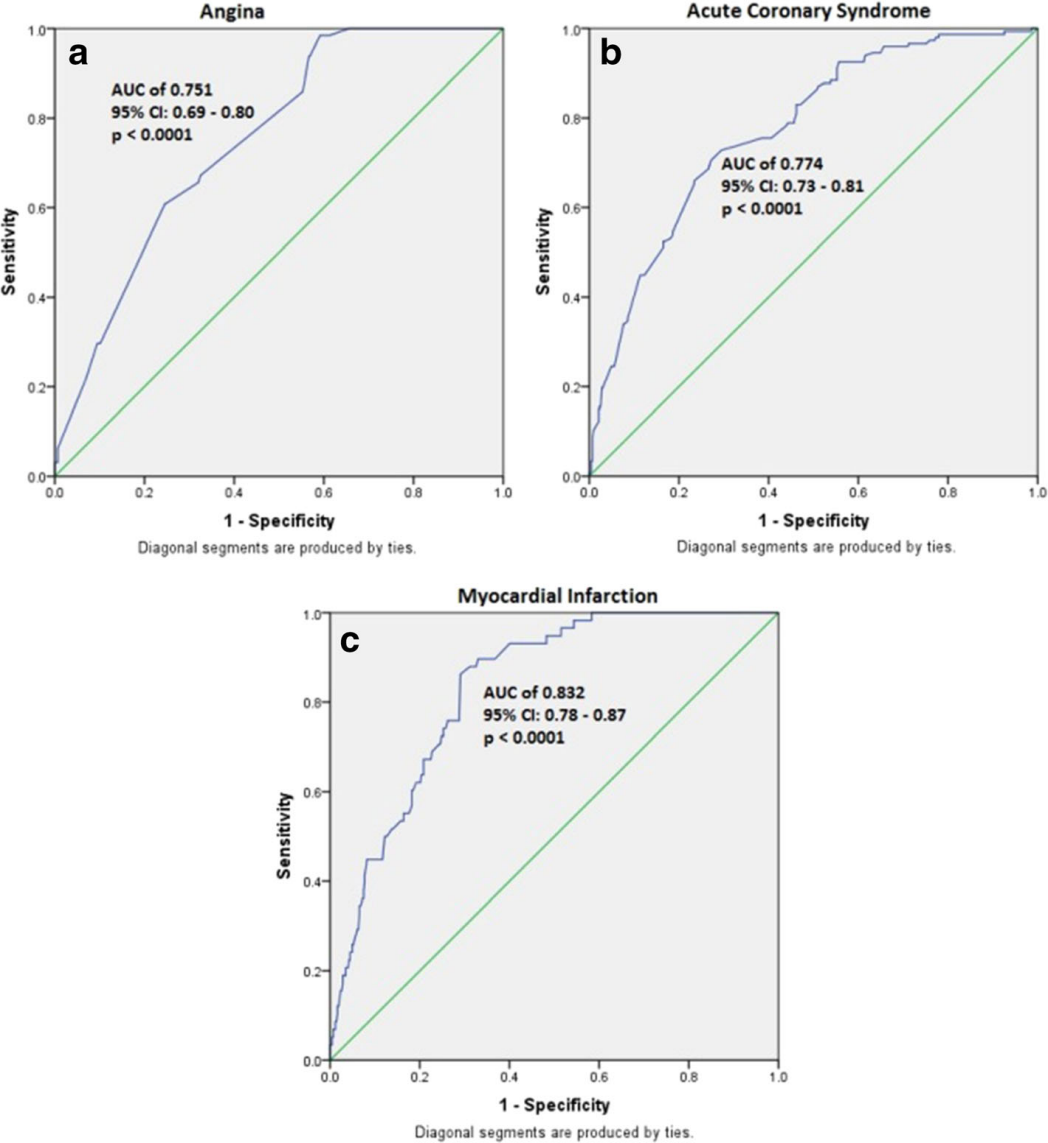
ROC analysis indicating discriminative power of the variants for phenotypic severity categories of CAD. **a** Angina. **b** Acute coronary syndrome. **c** Myocardial infarction

In order to determine the combined risk effect of the associated SNPs towards each of the anatomical and phenotypic categories, we computed the weighted mean proportion of the risk alleles of the SNPs by taking 2 for two risk alleles, 1 for one risk allele, and 0 for no risk alleles with weights as relative log odds ratios of different SNPs. The cumulative risk allele score for each individual is obtained by multiplying with number of SNPs associated with the clinical category. The risk scores for individuals ranged from 1.5 to 12.5, 0.5 to 15.5, 0.5 to 18.5, and 5.5 to 17.5 in case of insignificant, SVD, DVD, and TVD anatomical conditions of CAD, respectively. Similarly, the ranges are 0 to 8.5, 0.5 to 14.5, and 3.5 to 18.5 in case of angina, ACS, and MI, respectively. We grouped the risk scores with very low frequencies into the adjacent category. The details of risk categories and the risk scores constituting each of those categories are furnished in (Additional file 5: Table S5) and (Additional file 6: Table S6), according to each of the anatomical and phenotypic severity conditions. The results suggest a clear trend of increase in the proportion of cases with increasing risk score. With reference to baseline category with lowest risk scores, we computed odds ratios for each of the remaining risk categories. An increasing trend of OR values with increasing risk score is apparent with all the clinical conditions of CAD (Additional files 7 and 8). Except for the risk category 1 of ACS, the odds ratios suggest significant associations with clinical categories of CAD. To gauge discriminative power of the risk scores, we constructed the ROC (receiver operating curve) plot (Figs. 1 and 2) for the risk scores and status of the clinical categories of CAD. The observed area under curve (AUC) indicates that this study has substantial and significant power to confer these genetic variants as predictors for clinical conditions of CAD.

## Conclusions

The intrinsic biological processes and methodological aspects have always been confounding factors for studying complex diseases, which led to inconsistent results in genetic studies based on candidate gene and genome wide approaches [29, 30]. Although several attempts were made to minimize the confounding effects of these factors, the genetic background of characteristic clinical heterogeneity of CAD is not well addressed until recently [7]. From the results of our study, it may be hypothesized that a complex interaction between the intronic domains of regulatory genes and intergenic protein coding genes at 11q23.3 chromosomal region is involved in regulating the lipid traits. The high discriminative power of the variants further implicates their significant role in developing various forms of CAD. Given the relatively smaller sample sizes for the sub phenotypes, the statistical power for our study is somewhat limited and hence this study can be considered only exploratory in nature. Nevertheless, because of the paucity of such studies, we still believe that our results would provide useful insights into the possible genetic mechanisms behind the phenotypic heterogeneity of CAD, which might help in aptly designing future studies based on much larger samples.

## Additional files

Additional file 1: Table S1. Baseline characteristics of the controls and anatomical categories of CAD and the *p* values (*t* test) for mean difference between controls and each of the anatomical categories. (DOCX 13 kb)
Additional file 2:Table S2. Baseline characteristics of the controls and phenotypic severity categories of CAD and the *p* values (*t* test) for mean difference between controls and each of the phenotypic severity categories. (DOCX 12 kb)
Additional file 3: Table S3. Genotypic odds ratios for the SNPs significantly associated under log-additive model with anatomical categories. (DOCX 15 kb)
Additional file 4: Table S4. Genotypic odds ratios for the SNPs significantly associated under log-additive model with phenotypic severity. (DOCX 13 kb)
Additional file 5: Table S5. Groups of risk scores for anatomical categories. (DOCX 11 kb)
Additional file 6: Table S6. Groups of risk scores for phenotypic severity categories. (DOCX 11 kb)
Additional file 7: Figure S1. Plot of odds ratios for risk categories of different anatomical categories of CAD. (JPG 106 kb)
Additional file 8: Figure S2. Plot of odds ratios for risk categories of different phenotypic severity categories of CAD. (JPG 75 kb)
