# Supplementary material for: Genetic determinants of clinical heterogeneity of the coronary artery disease in the population of Hyderabad, India
Source: Hum Genomics. 2017 Mar 4;11:3. doi: 10.1186/s40246-017-0099-1 (PMC5336666; doi:10.1186/s40246-017-0099-1)
Supplement: Additional file 1: Table S1. — Baseline characteristics of the controls and anatomical categories of CAD and the p values (t test) for mean difference between controls and each of the anatomical categories. (DOCX 13 kb) [file 40246_2017_99_MOESM1_ESM.docx]

**Table S1 Baseline characteristics of the controls and anatomical categories of CAD and the p-values (t-test) for mean difference between controls and each of the anatomical categories**

| **Variable** | **Controls (n=462)** | **Insignificant (n=93)** | | **SVD (n=121)** | | **DVD (n=75)** | | **TVD (n=70)** | |
| --- | --- | --- | --- | --- | --- | --- | --- | --- | --- |
|  | **Mean ± SD** | **Mean ± SD** | **p value** | **Mean ± SD** | **p value** | **Mean ± SD** | **p value** | **Mean ± SD** | **p value** |
| **Age** | 50.74 **±** 9.8 | 54.0 ± 11.0 | 0.008* | 53.8 ± 10.7 | 0.005* | 55.0 ± 9.2 | 0.001* | 59.6 ± 8.7 | 0.001* |
| **BMI** | 26.9 **±** 4.5 | 26.7 ± 4.3 | 0.85 | 26.0 ± 4.0 | 0.16 | 26.0 ± 4.0 | 0.24 | 25.4 ± 2.7 | 0.009* |
| **FBS** | 96.5 **±** 41.0 | 141.5 ± 56.0 | 0.001* | 159.2 ± 63.2 | 0.001* | 167.0 ± 64.5 | 0.001* | 159.4 ± 55.3 | 0.001* |
| **Height** | 158.6 ± 9.2 | 159.0 ± 8.8 | 0.57 | 162.2 ± 7.0 | 0.001* | 163.8 ± 6.8 | 0.001* | 161.9 ± 6.9 | 0.011* |
| **Weight** | 67.6 ± 13.5 | 67.8 ± 11.3 | 0.86 | 68.4 ± 10.4 | 0.54 | 69.9 ± 11.2 | 0.22 | 66.8 ± 8.6 | 0.65 |
| **SBP** | 127.3 ± 14.5 | 130.1 ± 15.8 | 0.25 | 130.8 ± 15.5 | 0.11 | 133.0 ± 18.9 | 0.07 | 131.8 ± 18.4 | 0.19 |
| **DBP** | 83.4 ± 9.2 | 80.8 ± 9.6 | 0.06 | 82.0 ± 9.6 | 0.26 | 81.7 ± 10.7 | 0.33 | 81.4 ± 13.3 | 0.38 |
| **TC** | 190.6 ± 38.7 | 148.7 ± 23 | 0.001* | 156.4 ± 34.5 | 0.001* | 149.2 ± 30.2 | 0.001* | 154.2 ± 43.3 | 0.001* |
| **TG** | 161± 107 | 130.0 ± 73.2 | 0.001* | 155.9 ± 82.0 | 0.53 | 143.0 ± 73.8 | 0.07 | 147.5 ± 88.0 | 0.239 |
| **HDLC** | 47.7 ± 30.5 | 41.0 ± 2.0 | 0.001* | 41.3 ± 2.4 | 0.001* | 41.2 ± 2.3 | 0.001* | 41.3 ± 2.1 | 0.001* |
| **LDLC** | 113.6 ± 33.3 | 81.4 ± 19.3 | 0.001* | 84.8 ± 28.3 | 0.001* | 82.6 ± 24.9 | 0.001* | 86.5 ± 38.7 | 0.001* |
| **VLDL** | 32.1 ± 21.4 | 27.0 ± 19.2 | 0.028* | 31.0 ± 16.5 | 0.50 | 29.1 ± 15.7 | 0.15 | 27.8 ± 18.8 | 0.08 |

BMI – Body Mass Index, SBP – Systolic Blood Pressure, DBP – Diastolic Blood Pressure, FBS – Fasting Blood Sugar, TC – Total Cholesterol, TG – Triglycerides, HDLC – High Density Lipoprotein Cholesterol, LDLC – Low Density Lipoprotein Cholesterol, VLDL – Very Low Density Lipoprotein

*Significant
