# Supplementary material for: Genetic determinants of clinical heterogeneity of the coronary artery disease in the population of Hyderabad, India
Source: Hum Genomics. 2017 Mar 4;11:3. doi: 10.1186/s40246-017-0099-1 (PMC5336666; doi:10.1186/s40246-017-0099-1)
Supplement: Additional file 2:Table S2. — Baseline characteristics of the controls and phenotypic severity categories of CAD and the p values (t test) for mean difference between controls and each of the phenotypic severity categories. (DOCX 12 kb) [file 40246_2017_99_MOESM2_ESM.docx]

**Table S2 Baseline characteristics of the controls and phenotypic severity categories of CAD and the p-values (t-test) for mean difference between controls and each of the phenotypic severity categories**

| **Variable** | **Controls (n=462)** | **Angina (n=73)** | | **ACS (n=165)** | | **MI (n=76)** | |
| --- | --- | --- | --- | --- | --- | --- | --- |
|  | **Mean ± SD** | **Mean ± SD** | **p value** | **Mean ± SD** | **p value** | **Mean ± SD** | **p value** |
| **Age** | 50.74 **±** 9.8 | 56.9 ± 9.0 | 0.001* | 55.4 ± 10.6 | 0.001* | 54.2 ± 9.9 | 0.005* |
| **BMI** | 26.9 **±** 4.5 | 26.0 ± 3.9 | 0.17 | 26.3 ± 4.3 | 0.28 | 25.3 ± 3.3 | 0.012* |
| **FBS** | 96.5 **±** 41.0 | 161.6 ± 63.2 | 0.001* | 162.0 ± 61.3 | 0.001* | 147.8 ± 57.5 | 0.001* |
| **Height** | 158.6 ± 9.2 | 162.6 ± 8.7 | 0.007* | 161.4 ± 7.8 | 0.004* | 162.3 ± 6.7 | 0.002* |
| **Weight** | 67.6 ± 13.5 | 68.7 ± 10.7 | 0.53 | 68.5 ± 11.3 | 0.49 | 66.9 ± 10.2 | 0.72 |
| **SBP** | 127.3 ± 14.5 | 127.3 ± 15.8 | 0.97 | 133.7 ± 17.1 | 0.002* | 132.7 ± 19.6 | 0.12 |
| **DBP** | 83.4 ± 9.2 | 81.8 ± 9.2 | 0.29 | 81.9 ± 12.1 | 0.27 | 81.2 ± 9.6 | 0.17 |
| **TC** | 190.6 ± 38.7 | 147.1 ± 28.2 | 0.001* | 153.8 ± 33.8 | 0.001* | 155.7 ± 36.4 | 0.001* |
| **TG** | 161± 107 | 134.2 ± 65.8 | 0.004* | 154.5 ± 91.5 | 0.41 | 137.1 ± 66.8 | 0.011* |
| **HDLC** | 47.7 ± 30.5 | 41.2 ± 1.8 | 0.001* | 41.3 ± 2.2 | 0.001* | 41.1 ± 1.9 | 0.001* |
| **LDLC** | 113.6 ± 33.3 | 79.7 ± 23.6 | 0.001* | 84.6 ± 29.4 | 0.001* | 87.0 ± 29.2 | 0.001* |
| **VLDL** | 32.1 ± 21.4 | 28.6 ± 19.1 | 0.14 | 30.7 ± 18.8 | 0.39 | 26.5 ± 14.1 | 0.004* |

BMI – Body Mass Index, SBP – Systolic Blood Pressure, DBP – Diastolic Blood Pressure, FBS – Fasting Blood Sugar, TC – Total Cholesterol, TG – Triglycerides, HDLC – High Density Lipoprotein Cholesterol, LDLC – Low Density Lipoprotein Cholesterol, VLDL – Very Low Density Lipoprotein

*Significant
