# Supplementary material for: Genetic determinants of clinical heterogeneity of the coronary artery disease in the population of Hyderabad, India
Source: Hum Genomics. 2017 Mar 4;11:3. doi: 10.1186/s40246-017-0099-1 (PMC5336666; doi:10.1186/s40246-017-0099-1)
Supplement: Additional file 3: Table S3. — Genotypic odds ratios for the SNPs significantly associated under log-additive model with anatomical categories. (DOCX 15 kb) [file 40246_2017_99_MOESM3_ESM.docx]

**Table S3 Genotypic odds ratios for the SNPs significantly associated under log additive model with anatomical categories.**

| **SNP** | **Nearby/ associated Gene** | **Insignificant**  **(n = 93)** | | **Single Vessel Disease**  **(n = 121)** | | **Double Vessel Disease**  **(n = 75)** | | **Triple Vessel Disease**  **(n = 70)** | |
| --- | --- | --- | --- | --- | --- | --- | --- | --- | --- |
|  |  | **p value** | **OR (95% CI)** | **p value** | **OR (95% CI)** | **p value** | **OR (95% CI)** | **p value** | **OR (95% CI)** |
| **rs17440396:G>A** | BUD13 | 1.5x10^-13^ | 0.05  (0.02 - 0.17) | 3.2x10^-13^ | 0.11  (0.05 - 0.23) | 6.5x10^-10^ | 0.12  (0.05 - 0.29) | 2.5x10^-07^ | 0.13  (0.05 - 0.32) |
| **rs10488699:G>A** |  | 0.046 | 1.48  (1.01 - 2.18) |  |  |  |  | 0.052 | 1.54  (1.00 - 2.37) |
| **rs664059:C>T** |  |  |  | 0.029 | 1.37  (1.03 - 1.81) |  |  | 0.053 | 1.42  (1.00 - 2.03) |
| **rs6589566:A>G** | ZPR1 | 0.0001 | 2.00  (1.45 - 2.75) | 0.0007 | 1.65  (1.24 - 2.20) | 0.0001 | 1.95  (1.39 - 2.74) | 0.004 | 1.75  (1.20 - 2.55) |
| **rs3741298:A>G** |  | 0.052 | 0.72  (0.51 - 1.01) |  |  |  |  |  |  |
| **rs2075294:G>T** |  |  |  | 0.019 | 1.87  (1.13 - 3.12) |  |  | 0.070 | 1.82  (0.98 - 3.38) |
| **rs633389:C>T** | APOA5-APOA4 | 0.011 | 0.53  (0.31 - 0.90) | 0.030 | 0.63  (0.40 - 0.98) | 0.0003 | 0.32  (0.16 - 0.67) | 0.003 | 0.40  (0.20 - 0.80) |
| **rs633867:C>T** |  |  |  | 0.015 | 1.89  (1.14 - 3.12) |  |  |  |  |
| **rs11600380:T>C** |  |  |  |  |  |  |  | 0.026 | 0.63  (0.42 - 0.96) |
| **rs1263163:G>A** |  |  |  |  |  | 0.018 | 0.44  (0.25 - 0.78) | 0.011 | 0.40  (0.22 - 0.74) |
| **rs625524:G>A** |  | 0.008 | 2.34  (1.28 - 4.27) |  |  |  |  | 0.007 | 0.14  (0.02 - 1.03) |
| **rs1263171:G>A** |  |  |  |  |  | 0.024 | 1.46  (1.05 - 2.04) | 0.010 | 1.56  (1.11 - 2.21) |
| **rs2727793:G>A** |  |  |  |  |  | 0.042 | 1.41  (1.01 - 1.97) |  |  |
| **rs7396835:C>T** |  |  |  |  |  | 0.071 | 0.73  (0.52 - 1.03) |  |  |
| **rs2542063:G>A** |  |  |  |  |  | 0.048 | 1.41  (1.00 - 1.97) |  |  |
| **rs2849165:G>A** |  | 2.5x10^-05^ | 0.46  (0.31 - 0.67) | 1.9x10^-05^ | 0.49  (0.35 - 0.69) | 0.0003 | 0.48  (0.32 - 0.74) | 0.004 | 0.55  (0.36 - 0.84) |
| **rs5081:A>T** | APOA1 |  |  | 0.028 | 1.95  (1.10 - 3.47) |  |  |  |  |
| **rs5072:C>T** |  |  |  | 0.043 | 0.74  (0.55 - 1.00) |  |  |  |  |
| **rs632153:G>T** |  |  |  | 0.046 | 2.00  (1.05 - 3.80) |  |  |  |  |

Blank cell – Not significant, OR-Odds ratio obtained from logistic regression analysis
