# Supplementary material for: Genetic determinants of clinical heterogeneity of the coronary artery disease in the population of Hyderabad, India
Source: Hum Genomics. 2017 Mar 4;11:3. doi: 10.1186/s40246-017-0099-1 (PMC5336666; doi:10.1186/s40246-017-0099-1)
Supplement: Additional file 4: Table S4. — Genotypic odds ratios for the SNPs significantly associated under log-additive model with phenotypic severity. (DOCX 13 kb) [file 40246_2017_99_MOESM4_ESM.docx]

**Table S4 Genotypic odds ratios for the SNPs significantly associated under log additive model with phenotypic severity.**

| **SNP** | **Nearby/ associated Gene** | **Angina**  **(n = 93)** | | **Acute Coronary Syndrome**  **(n = 121)** | | **Myocardial Infarction**  **(n = 75)** | |
| --- | --- | --- | --- | --- | --- | --- | --- |
|  |  | **p value** | **OR (95% CI)** | **p value** | **OR (95% CI)** | **p value** | **OR (95% CI)** |
| **rs17440396:G>A** | BUD13 | 2.6x10^-11^ | 0.05  (0.01 - 0.19) | 7.8x10^-19^ | 0.10  (0.05 - 0.20) | 1.1x10^-09^ | 0.09  (0.03 - 0.25) |
| **rs10488699:G>A** |  |  |  |  |  | 1.5x10^-04^ | 2.26  (1.49 - 3.43) |
| **rs664059:C>T** |  |  |  | 0.043 | 1.30  (1.01 – 1.67) | 0.011 | 1.54  (1.10 - 2.16) |
| **rs2187126:A>G** |  |  |  | 0.019 | 0.60  (0.39 - 0.94) |  |  |
| **rs6589566:A>G** | ZPR1 | 6.6x10^-06^ | 2.30  (1.60 - 3.29) | 2.5x10^-08^ | 2.06  (1.59 - 2.66) |  |  |
| **rs2075294:G>T** |  |  |  | 0.067 | 1.57  (0.98 - 2.52) | 0.042 | 1.90  (1.05 - 3.41) |
| **rs633389:C>T** | APOA5-APOA4 | 0.012 | 0.38  (0.19 - 0.75) | 0.011 | 0.62  (0.42 - 0.91) | 0.022 | 0.43  (0.22 - 0.84) |
| **rs633867:C>T** |  |  |  | 0.011 | 1.77  (1.15 - 2.73) |  |  |
| **rs1263163:G>A** |  |  |  |  |  | 3.4x10^-09^ | 0.09  (0.03 - 0.26) |
| **rs1263167:A>G** |  | 0.055 | 1.63  (1.01 - 2.64) |  |  |  |  |
| **rs1263171:G>A** |  |  |  |  |  | 0.049 | 1.39  (1.00 - 1.93) |
| **rs2849165:G>A** |  |  |  | 3.3x10^-11^ | 0.36  (0.26 - 0.50) | 0.001 | 0.52  (0.34 - 0.79) |
| **rs2849176:G>C** | APOA1 |  |  |  |  | 0.031 | 1.45  (1.03 - 2.05) |
| **rs5081:A>T** |  |  |  |  |  | 0.045 | 2.11  (1.06 - 4.23) |
| **rs632153:G>T** |  |  |  |  |  | 0.053 | 2.21  (1.03 - 4.75) |

Blank cell – Not significant, OR-Odds ratio obtained from logistic regression analysis
