# Supplementary material for: Genetic determinants of clinical heterogeneity of the coronary artery disease in the population of Hyderabad, India
Source: Hum Genomics. 2017 Mar 4;11:3. doi: 10.1186/s40246-017-0099-1 (PMC5336666; doi:10.1186/s40246-017-0099-1)
Supplement: Additional file 5: Table S5. — Groups of risk scores for anatomical categories. (DOCX 11 kb) [file 40246_2017_99_MOESM5_ESM.docx]

**Table S5 Groups of risk scores for anatomical categories**

| **RISK CATEGORY** | **Risk Score Groups for Anatomical Categories** | | | |
| --- | --- | --- | --- | --- |
|  | **Insignificant** | **SVD** | **DVD** | **TVD** |
| Baseline | 1.5 to 8.5 | 0.5 to 8.5 | 0.5 to 10.5 | 5.5 to 13.5 |
| Category 1 | 8.6 to 9.5 | 8.6 to 9.5 | 10.6 to 11.5 | 13.6 to 14.5 |
| Category 2 | 9.6 to 10.5 | 9.6 to 10.5 | 11.6 to 12.5 | 14.6 to 15.5 |
| Category 3 | 10.6 to 11.5 | 10.6 to 11.5 | 12.6 to 13.5 | 15.6 to 16.5 |
| Category 4 | 11.6 to 12.5 | 11.6 to 15.5 | 13.6 to 18.5 | 16.6 to 17.5 |
