# Supplementary figures and images for: Genetic determinants of clinical heterogeneity of the coronary artery disease in the population of Hyderabad, India
Source: Hum Genomics. 2017 Mar 4;11:3. doi: 10.1186/s40246-017-0099-1 (PMC5336666; doi:10.1186/s40246-017-0099-1)

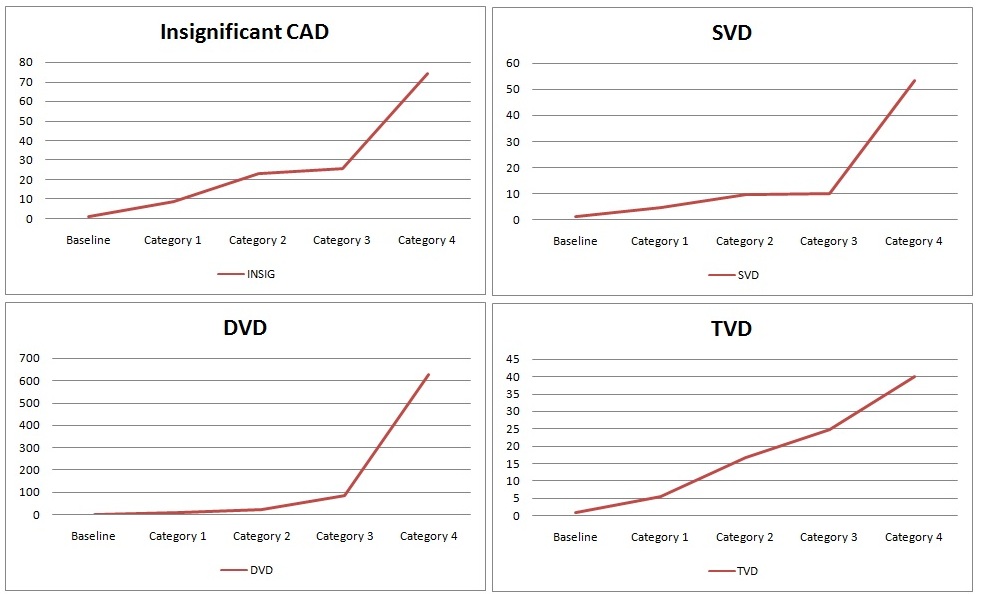

Supplement: Additional file 7: Figure S1. — Plot of odds ratios for risk categories of different anatomical categories of CAD. (JPG 106 kb) [file 40246_2017_99_MOESM7_ESM.jpg]

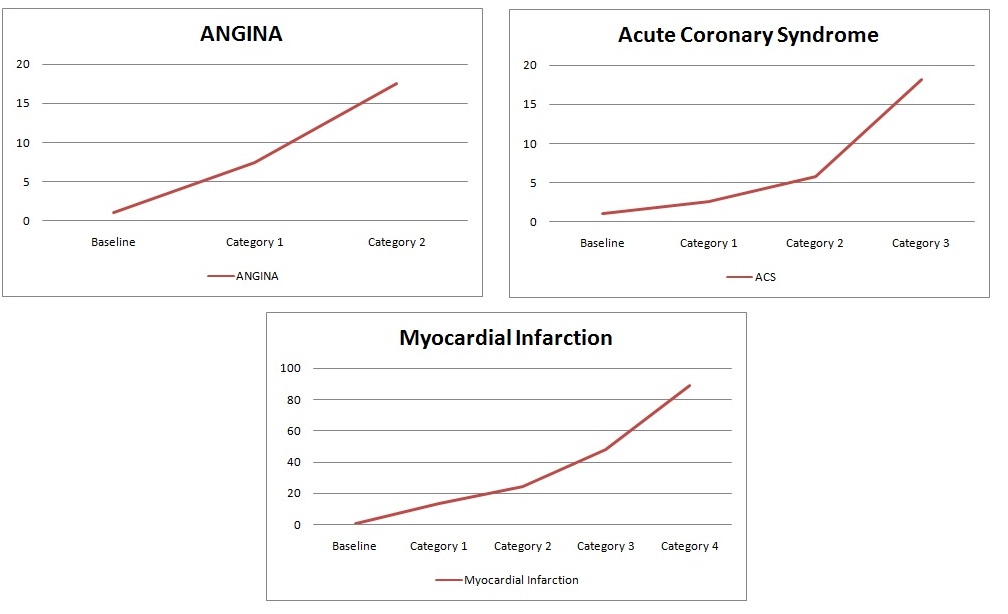

Supplement: Additional file 8: Figure S2. — Plot of odds ratios for risk categories of different phenotypic severity categories of CAD. (JPG 75 kb) [file 40246_2017_99_MOESM8_ESM.jpg]
